# The Avian Transcription Factor c-Rel is Expressed in Lymphocyte Precursor Cells and Antigen-Presenting Cells During Thymus Development

**DOI:** 10.1155/1998/58608

**Published:** 1998

**Authors:** C. Huguet, F. Bouali, P. J. Enrietto, D. Stehelin, B. Vandenbunder, C. Abbadie

**Affiliations:** 1 Laboratoire d'Oncologie Moléculaire URA1160 Institut de Biologie de Lille 1 rue Calmette Lille Cedex 59019 France; 2 Department of Microbiology State University of New York at Stony Brook New York 11794 USA

**Keywords:** Rel/NF-κB, thymus, lymphocyte precursor cells, antigen-presenting cells, avian development

## Abstract

Transcription factors of the Rel/NF-*κ*B family are widely involved in the immune system. In this study, we investigate the *in vivo* expression of the avian protein c-Rel in the T-cell lineage during thymus development. The majority of thymocytes do not express the c-Rel protein. However, lymphocyte precursor cells that colonize the thymus express the c-Rel protein shortly after their homing in the organ and before they begin to differentiate, c-Rel is also detected in different subsets of,antigen-presenting cells such as epithelial cells, dendritic cells, and macrophages. *In vitro* studies have shown that Rel/NF-*κ*B proteins are sequestered in an inactive form in the cytoplasm by interaction with the I*κ*B*α* inhibitory protein. By immunocytochemistry, we show that *in vivo* c-Rel is localized in the cytoplasm of antigen-presenting cells but in both the cytoplasm and nucleus of lymphocyte precursor cells. The cytoplasmic localization of c-Rel in antigen-presenting cells correlates with a high expression of I*κ*B*α*, whereas the nuclear localization of c-Rel in lymphocyte precursor cells correlates with a much lower expression of I*κ*B*α*. These results suggest that c-Rel might be constitutively activated in lymphocyte precursor cells.


*Developmental Immunology*, 1998, Vol. 5, pp. 247–261
Reprints available directly from the publisher
Photocopying permitted by license only

© 1998 OPA (Overseas Publishers Association)
N.V. Published by license under the
Harwood Academic Publishers imprint,
part of the Gordon and Breach Publishing Group
Printed in Malaysia

### The Avian Transcription Factor c-Rel Is Expressed in Lymphocyte Precursor Cells and Antigen-Presenting Cells During Thymus Development

C. HUGUET[a], F. BOUALI[a], P.J. ENRIETTO[b], D. STEHELIN[a], B. VANDENBUNDER[a] and C. ABBADIE[a*]

[a]*Laboratoire d'Oncologie Moléculaire, URA1160, Institut de Biologie de Lille, 1 rue Calmette, 59019 Lille Cedex, France;* [b]*Department of Microbiology, State University of New York at Stony Brook, New York 11794*

*(Received 6 January 1997; Revised 5 June 1997; In final form 18 June 1997)*

Transcription factors of the Rel/NF-κB family are widely involved in the immune system. In this study, we investigate the *in vivo* expression of the avian protein c-Rel in the T-cell lineage during thymus development. The majority of thymocytes do not express the c-Rel protein. However, lymphocyte precursor cells that colonize the thymus express the c-Rel protein shortly after their homing in the organ and before they begin to differentiate. c-Rel is also detected in different subsets of antigen-presenting cells such as epithelial cells, dendritic cells, and macrophages. *In vitro* studies have shown that Rel/NF-κB proteins are sequestered in an inactive form in the cytoplasm by interaction with the IκBα inhibitory protein. By immunocytochemistry, we show that *in vivo* c-Rel is localized in the cytoplasm of antigen-presenting cells but in both the cytoplasm and nucleus of lymphocyte precursor cells. The cytoplasmic localization of c-Rel in antigen-presenting cells correlates with a high expression of IκBα, whereas the nuclear localization of c-Rel in lymphocyte precursor cells correlates with a much lower expression of IκBα. These results suggest that c-Rel might be constitutively activated in lymphocyte precursor cells.

*Keywords:* Rel/NF-κB, thymus, lymphocyte precursor cells, antigen-presenting cells, avian development

### INTRODUCTION

The proto-oncogene *c-rel* encodes a transcription factor of the Rel/NF-κB family. The members of this family share a highly conserved 300 amino acid N-terminal domain, the Rel Homology Domain (RHD), involved in DNA binding, dimerization, nuclear localization, and interaction with inhibitory proteins of the IκB family. Rel/NF-κB proteins associate as homo- or heterodimers and bind a specific DNA sequence, the κB motif, in the promoter of their target genes. Inside the Rel/NF-κB family, two subfamilies of proteins can be defined based on the function of their C-terminal domains. The first one includes c-Rel, RelA, RelB, Dorsal, and Dif, which contain a transactivating domain in their C terminus. The

*Corresponding author.

second family is composed of the precursor proteins NF-κB1 and NF-κB2, which contain IκB-like ankyrin repeats in their C terminus. NF-κB1 and NF-κB2 undergo a proteolytical processing that eliminates their ankyrin motifs and generates proteins p50 and p52, respectively. These mature p50 and p52 are able to homo- or heterodimerize with other members of the Rel/NF-κB family, thus becoming active transcription factors (reviewed in Miyamoto and Verma, 1995; Verma et al., 1995).

In most cell types, Rel/NF-κB dimers are found in an inactive form sequestered in the cytoplasm by an ankyrin repeat protein of the IκB family. The IκB family is composed of IκBα, IκBβ, and Bcl3, as well as NF-κB1 and NF-κB2 precusors in their unprocessed form. The activation of the Rel/NF-κB proteins is regulated by a posttranslational mechanism, which includes the phosphorylation of the IκB inhibitor and its subsequent degradation, thus liberating the Rel/NF-κB dimer, which can translocate into the nucleus (reviewed in Verma et al., 1995; Miyamoto and Verma, 1995). The inhibitor degradation occurs within minutes after stimulation, making the Rel/NF-κB transcription factors very efficient when rapid responses are needed. The immune system takes advantage of this property and extensively uses the Rel/NF-κB proteins for regulating cytokine, growth factor, acute phase protein, and immunoreceptor gene expression (review in Bauerle and Henkel, 1994; Kopp and Ghosh, 1995). The Rel/NF-κB proteins are also involved in pathogenesis of the immune system. In humans, alterations at the *rel* locus have been found in follicular and diffuse large cell lymphomas (Lu et al., 1991) and amplifications of the c-*rel* gene have been detected in primary mediastinal thymic B-cell lymphoma (Joos et al., 1996) and in extranodal diffuse large cell lymphoma (Houldsworth et al., 1996).

The expression of c-Rel has been widely described in the hematopoietic organs during mouse development. In the fetal liver, the c-Rel protein expression is restricted to hematopoietic precursor cells of the erythroid and B-cell lineage. In the spleen, c-Rel expression is also found in B cells. In the embryonic thymus, c-Rel is essentially detected in medullary epithelial cells and in some B cells, whereas thymocytes do not express any c-Rel protein. In the T-cell lineage, only mature helper and cytotoxic T cells of the lymph nodes express c-Rel (Carrasco et al., 1994). A better understanding of the mouse c-Rel functions in hematopoiesis and immune system was obtained with the establishment of null mice (Köntgen et al., 1995). In these mice, no developmental defect in any hematopoietic lineage could be evidenced, indicating that c-Rel is not essential for embryonic hematopoiesis. However, c-*rel*-deficient mice displayed defects in humoral immunity and unresponsiveness of mature B and T cells to most mitogenic stimuli.

In the avian embryo, such exhaustive studies have not yet been performed. Nevertheless previous *in vitro* analysis suggested a potential involvement of some Rel/NF-κB members in avian hematopoiesis (reviewed in Huguet et al., 1994). Northern blot analysis revealed that c-*rel* is predominantly expressed in chick hematopoietic organs such as the bursa of Fabricius, the thymus, the spleen, and the bone marrow (Moore and Bose, 1989). Moreover, the avian retrovirus Rev-T, bearing the v-Rel oncogene, causes fatal lymphomatosis in chicken (Sevoian et al., 1964; Olson, 1967). The *in vivo* preferential target cells for Rev-T are immature and mature lymphoid cells expressing B- and T-cell determinants as well as myeloid cells (Lewis et al., 1981; Barth et al., 1990; Zhang et al., 1991). *In vitro* studies also showed that the majority of v-Rel transformed hematopoietic cells bore lymphoid markers (Morrison et al., 1991). More recently, a v-RelER fusion protein has been shown to transform an early progenitor of dendritic and neutrophil cells (Boehmelt et al., 1995).

Altogether, these results prompted us to further investigate the relationships between c-Rel and hematopoiesis in chicken. We focused our study on the embryonic thymus that constitutes the primary site of T-cell development. Since the activity of the avian c-Rel is tightly dependent on interactions with the IκBα inhibitor (Morrison et al., 1989; Davis et al., 1990; Kerr et al., 1991; Kochel et al., 1991; Hrdlickova et al., 1995; Schatzle et al., 1995), we studied in parallel the expression of both genes and searched for the nucleocytoplasmic localization of the c-Rel protein, which gives evidence about its activation.

### RESULTS

### High Expression of c-*rel* in the Thymus Correlates with the Differentiation of the Medulla

The analysis of c-*rel* expression was performed at three stages representative of the avian thymus development: (1) at E9.5, the thymus is an epithelio-mesenchymal rudiment filled up with very immature precursor cells; (2) at E15, small medullary areas are separating from the cortex, figuring the maturation of the thymic stroma; (3) at E19, the avian thymic structure is almost completed with well-defined cortical lobules and large medullary areas. The cortex is essentially composed of double-positive $CD4^{+}CD8^{+}$ immature thymocytes enclosed in an epithelial meshwork, whereas the medulla is mainly composed of single-positive mature thymocytes among antigen-presenting cells and epithelial cells.

*In situ* hybridization experiments performed at E9.5 show that c-*rel* mRNAs are homogeneously expressed throughout the thymic organ [Figures 1(A) and 1(B)]. At E15, c-*rel* expression becomes heterogeneous with higher levels in the developing medulla than in the cortex [Figures 1(C) and 1(D)]. At E19, this heterogeneous distribution of c-*rel* mRNAs is even more accentuated [Figures 1(E) and 1(F)]. At both E15 and E19 stages, high magnifications show that inside the medulla the areas of highest expression correspond to cells with large cytoplasm and nucleus that could be clusters of dendritic or epithelial cells and macrophages, according to their morphology and localization. In contrast, cortical and medullary thymocytes, recognizable by a small round nucleus and a low cytoplasmic volume, express lower levels of c-*rel* mRNAs (Figure 2). Thus, during the thymus development, the high expression of c-*rel* correlates with the establishment of mature medullary areas, whereas the expression in developing thymocytes is continuous and at low levels.

### The c-Rel Protein Is Expressed in Endothelial, Epithelial, and Antigen-Presenting Cells of the Thymus

To investigate the c-*rel* expression at the protein level, Western blot experiments were performed with an immuno-purified anti-c-Rel antibody on hematopoietic organs at different developmental stages. One major band at 68 kD, corresponding to the avian c-Rel protein molecular mass (Simek and Rice, 1988), is detected in the thymus at E15 and E19, although less intensely than in the spleen and the bursa of Fabricius (Figure 3). Immunocytochemistry experiments were performed with this same anti-c-Rel antibody on serial thymus sections to identify the c-Rel-expressing cells. At E15 and E19, consistent with its mRNA distribution, the c-Rel protein is predominantly expressed inside the medulla in large clusters of cells (Figure 4). Some isolated cells scattered in the cortex also express c-Rel. Cortical as well as medullary thymocytes, identified with anti-CD3, -CD4, and -CD8 antibodies (data not shown), do not express any detectable amounts of c-Rel. At least four different types of cells were found to express c-Rel. The most easily identifiable are endothelial cells bordering the thymic vessels [Figure 5(A)]. Epithelial cells of different morphology and localization, identified with an anti-cytokeratin antibody (data not shown), also express c-Rel: Some line the cortical lobules [Figure 5(B)] and others form large clusters inside the central medulla [Figure 5(C)]. Other medullary clusters, containing two to three large cells with large vacuoles, also express c-Rel. In these cells, the protein is clearly localized in the cytoplasm [Figure 5(D)]. These cells displayed the violet color characteristic of myeloid cells when stained by the panoptic method of Pappenheim (data not shown). According to their morphology, localization and staining properties, they could be dendritic cells. At the corticomedullary junction, inside the medulla and scattered inside the cortex, isolated large round cells also express c-Rel in their cytoplasm [Figure 5(E)]. These cells possess a characteristic eccentric nucleus that is clearly negative. We assume that these cells are macrophages because of their morphology, pink staining by the described in a mature mouse B-cell line and was explained by a high and continuous degradation of IκBα in the absence of external signals (Miyamoto et al., 1994). Here, we show that avian LPCs express low levels of IκBα, suggesting that the constitutive nuclear localization of c-Rel might be due to a low expression of the inhibitor. This hypothesis requires further investigation of IκBα expression at the protein level.

The involvement of c-Rel in proliferation has already been suggested by *in vitro* experiments showing that overexpression of c-Rel in chicken embryo fibroblasts led to morphological transformation, life-span extension, and proliferation (Abbadie et al., 1993; Kralova et al., 1994). The constitutive nuclear localization of c-Rel in LPCs documented in this paper represents the first *in vivo* argument in favor of a participation of this transcription factor in the proliferation of very immature progenitor of T cells.

### c-Rel Is Expressed in the Cytoplasm of Thymic Antigen-Presenting Cells

In avian thymuses from E15 to E19, c-Rel is expressed in epithelial cells, dendritic cells, and macrophages. Avian thymic epithelial cells, dendritic cells, and macrophages share common surface antigens and particularly MHC molecules (Peck et al., 1982; Guillemot et al., 1984; Boyd et al., 1992); they constitute the three main types of antigen-presenting cells (APCs) of the thymus. These cells govern positive and negative selection of T cells in the thymus, via interactions between their MHC and the T-cell receptor (TCR) (reviewed in von Boehmer, 1994; Nossal, 1994).

*In vitro* studies have shown that avian MHC class I and II genes are induced when c-Rel or v-Rel are overexpressed (Hrdlickova et al., 1994). Therefore, a potential function of c-Rel in APCs could be to regulate the expression of MHC genes. However, in macrophages, besides genes involved in T-cell maturation, c-Rel could regulate genes involved in basic antimicrobial functions, since in HD11 cells, a chicken myelomonocytic cell line, RelA, and c-Rel are able to activate the transcription of the lysozyme gene (Phi Van, 1996).

*In vitro* experiments have demonstrated that in chicken, IκBα is able to inactivate c-Rel by sequestering it in the cytoplasm (Davis et al., 1990; Kerr et al., 1991). In accordance with these studies, our *in situ* hybridization analysis revealed that *in vivo*, high expression of *ikba* is detected in all of the stromal cells that express the c-Rel protein in the cytoplasm, that is, in endothelial cells, epithelial cells, dendritic cells, and macrophages.

The expression pattern of c-Rel is more widespread in chick than in mouse APCs, where c-Rel appears restricted to medullary epithelial cells. Moreover, in mouse, the expression of c-Rel in progenitor cells is restricted to the erythroid and B-cell lineages (Carrasco et al., 1994). Our results on *ikba* are also different from those obtained in mouse. In the mouse thymus, *ikba* expression is higher in the cortex than in the medulla (Weih et al., 1994).

In conclusion, both avian and murine c-Rel proteins might be indirectly involved in T-cell maturation via their expression in antigen-presenting cells, but this expression seems restricted to epithelial cells in mouse, whereas it also extends to dendritic cells and macrophages in chicken. In contrast, the sole avian c-Rel protein might be also involved in early lymphopoiesis via its expression in proliferating lymphocyte precursor cells.

### MATERIAL AND METHODS

### Animals

Fertilized White Leghorn chicken eggs were incubated at 39°C in a humidified chamber. The age of embryos is indicated as E1, E2,..., E1 corresponding to 24 hr of incubation.

### Histological Sections

Chick embryo thymuses at different developmental stages were dissected in PBS and fixed at 4°C for 18 hr in 4% paraformaldehyde in PBS ($Na_2HPO_4$-$NaH_2PO_4$, 0.1 M, pH 7.4), then dehydrated in ethanol and toluene, embedded in paraffin, and processed for 5-6-$\mu$m histological sections.

### Isolation and Primary Culture of Macrophages and Dendritic Cells

The protocol was adapted from Oliver and Le Douarin (1984). E19 thymuses were dissected, minced with scissors, and crushed through a fine nylon gauze, which retains connective tissue and epithelial cells. The collected cell suspension was rinsed in culture medium (RPMI 1640, 10% FCS, 1000 IU/ml penicillin, 1000 $\mu$g/ml streptomycin) and resuspended in 10% BSA (Sigma) in RPMI 1640 buffered with Hepes pH 7.3. A discontinuous gradient of 22%, 27%, and 29% BSA in RPMI 1640, pH 7.3, was prepared in a 5-ml polyallomer tube and the cell suspension was layered on top of the gradient. After centrifugation at 35,000 g for 30 min, the fraction located above the 22% layer was collected. Cells enriched in macrophages and dendritic cells were rinsed in culture medium and plated on glass coverslips for 4 hr at 37°C in 5% $CO_2$ humidified incubator. Contaminating thymocytes were eliminated by hard pipetting. Adherent cells were fixed in 4% paraformaldehyde in PBS overnight at 4°C and processed for *in situ* hybridization or immunocytochemistry.

### *In Situ* Hybridization

#### Synthesis of $^{35}$S RNA Probes

The chicken full-length c-*rel* c-DNA was cloned in Bluescript SK$^-$ (Stratagene) (Abbadie et al., 1993). A 0.9-kb EcoRI fragment from the chicken *ikba* cDNA, kindly provided by H. Bose, was cloned in Bluescript SK$^-$ phagemid (Stratagene). $^{35}$S RNA probes were transcribed from 2 $\mu$g of linearized plasmids by 20 U of either T7, T3, or SP6 RNA polymerase for sense and antisense probes in a 20-$\mu$l reaction mixture containing 200 $\mu$Ci $^{35}$S-CTP (1300 Ci/mMol), 200 $\mu$M UTP, ATP, and GTP for 1 hr at 39°C. To facilitate their penetration into cells, probes were submitted to a limited alkaline hydrolysis generating fragments of approximately 150 bases as recommended by (Cox et al., 1984).

#### In Situ Hybridization

*In situ* hybridization was adapted from the method of Cox et al., (1984), as described by Quéva et al. (1992). Briefly, after being deparaffinized and rehydrated, sections were incubated in 0.1 M glycine, 0.2 M Tris-HCl, pH 7.4, for 10 min at 20°C, treated with 1 $\mu$g/ml proteinase K (Boehringer Mannheim) for 15 min at 37°C, postfixed in 4% paraformaldehyde, washed in PBS, acetylated 10 min with 0.25% acetic anhydride in 0.1 M triethanolamine, washed in 2X SSC (0.3 M NaCl, 30 mM sodium citrate), and dehydrated in ethanol. $^{35}$S RNA probes were denatured at 80°C and diluted in the hybridization buffer at a concentration of 50 pg/$\mu$l. Hybridization was performed at 60°C for 18 hr. After a wash in 4X SSC at 20°C, slides were treated with 10 $\mu$g/ml of RNase A (type III A, Sigma) for 30 min at 37°C, subsequently washed in 0.1X SSC at 60°C, dehydrated by ethanol, and dipped in nuclear track emulsion (Kodak NTB2). The sections were exposed at 4°C for 2 weeks. After developing, sections were stained with a DNA intercalating fluorescent dye (Hoechst 33258), and mounted and observed under dark-field and UV illumination. Both antisense and sense probes were used, and the sense probes never gave any signal.

### Immunocytochemistry and Western Blot

#### Antibodies

A rabbit immuno-purified anti-c-Rel serum (SB146) raised against the 15 carboxy terminal amino acids specific of the chicken c-Rel protein (Abbadie et al., 1993) was used both in Western blot and immunocytochemistry experiments. A normal rabbit immunoglobulin fraction (Dako) was used as negative control. A rabbit polyclonal anti-keratine serum (Dako) was used in immunocytochemistry to identify epithelial cells. Mouse monoclonal anti-chicken-CD3, -CD4, and -CD8 antibodies (Southern Biotechnology Associates) were used in immunocytochemistry to identify the different types of thymocytes.

### Immunocytochemistry

After being deparaffinized and rehydrated, sections were incubated in 0.5 mg/ml Saponine (Sigma) in PBS for 30 min; then endogenous peroxidase activity was quenched by incubation in 80% methanol, 20% PBS, 0.6% $H_2O_2$ for 30 min followed by 30 min saturation in 5% dry defatted milk in PBS. Sections were incubated with the primary antibody overnight at 4°C. After three washes in PBS, the EXTRA-3 kit (Sigma Immunochemicals) and the AEC substrate system (Dako) were used to detect the primary antibody. Sections were counterstained with hematoxylin (Sigma) and mounted in Glycergel (Dako).

### Western Blot of Hematopoietic Organs

Hematopoietic organs at various developmental stages were dissected in PBS, transferred into a solution containing 0.06 M tris-HCl, pH6.8, 5% $\beta$-mercaptoethanol, 2% SDS, 10% glycerol, 0.002% bromophenol blue, homogenized in a Dounce, sonicated and centrifuged at 10,000 g. In order to measure protein concentration in each sample, 20-$\mu$l aliquots were separated on a 10% SDS-PAGE; proteins were then stained with Coomassie-blue and the gel scanned lane by lane. Equicharged 10% SDS-PAGE were run and transferred onto nitrocellulose sheets. Blots were first incubated 30 min with 5% dry defatted milk in PBS, then 2 hr at room temperature with SB146. After three washes in PBS, the sheets were incubated with horseradish-peroxidase-conjugated swine anti-rabbit immunoglobulin (Dako) for 2 hr at room temperature. After washing, the peroxidase activity was revealed by incubation in diaminobenzidine (30 mg in 100 ml 0.05 M tris-HCl, pH 7.4, 0.01% $H_2O_2$).

### Panoptic Coloration of Pappenheim

This protocol of hematological coloration is described in Gabe (1968). Briefly, after being deparaffinized and rehydrated, sections were stained 3 min in May-Grünwald, 3 min in May-Grünwald/Sörensen buffer ($Na_2HPO_4$-$KH_2PO_4$, 0.1 M, pH 6.8) V/V, washed in Sörensen buffer, stained in Giemsa/Sörensen buffer 1V/75V for 15 min, washed in Sörensen buffer, differentiated in 0.15% acetic acid, washed in Sörensen buffer, and dehydrated in two washes of acetone and one wash of toluene. Sections were then mounted in Eukitt (O. Kindler GmbH, Germany).

### *Acknowledgements*

We are grateful to Eric Maire and Virginie Mattot for their technical help and advice. We thank Jean Coll, Pierre-Antoine Desfossez, Véronique Fafeur, Corine Glineur, and Fabrice Soncin for critical reading of the manuscript. This work was supported by grants from the Institut Pasteur de Lille, the Centre National de la Recherche Scientifique, and the Association pour la Recherche sur le Cancer. C.H. was supported by a training grant from the Association pour la Recherche sur le Cancer.

### *References*

Abbadie C., Kabrun N., Bouali F., Smardova J., Stéhelin D., Vandenbunder B. and Enrietto P. (1993). High levels of c-*rel* expression are associated with programmed cell death in the developing avian embryo and in bone marrow cells *in vitro*. Cell **75**: 899-912.

Barth C.F., Ewert D.L., Olson W.C. and Humphries E.H. (1990). Reticuloendotheliosis virus REV-T (REV-A)-induced neoplasia: Development of tumors within the T-lymphoid and myeloid lineages. J. Virol. **64**: 6054-6062.

Bauerle P.A. and Henkel T. (1994). Function and activation of NF-$\kappa$B in the immune systeme. Annu. Rev. Immunol. **12**: 141-179.

Boehmelt G., Madruga J., Dörfler P., Briegel K., Schwarz H., Enrietto P.J., and Zenke M. (1995). Dendritic cell progenitor is transformed by a conditional v-Rel Estrogen Receptor fusion protein v-RelER. Cell **80**: 341-352.

Boyd R.L., Wilson T.J., Bean A.G. and Ward H.A. (1992). Phenotypic characterization of chicken thymic stromal elements. Dev. Immunol. **2**: 51-66.

Carrasco D., Weih F. and Bravo R. (1994). Developmental expression of the mouse c-rel proto-oncogene in hematopoietic organs. Development **120**: 2991-3004.

Coltey M., Jotereau F.V. and Le Douarin N.M. (1987). Evidence for a cyclic renewal of lymphocyte precursor cells in the embryonic chick thymus. Cell Diff. **22**: 71-82.

Cox K.H., DeLeon D.V., Angerer L.M. and Angerer R.C. (1984). Detection of mRNAs in sea urchin embryos by *in situ*

hybridization using asymmetric RNA probes. Dev. Biol. **101**: 485-502.

Davis J.N., Bargmann W. and Bose H.J. (1990). Identification of protein complexes containing the c-rel proto-oncogene product in avian hematopoietic cells. Oncogene **5**: 1109-1115.

Dieterlen-Lièvre F. (1994). Hemopoiesis during avian Ontogeny. Poultry Sci. Rev. **5**: 273-305.

Duncan R.L. and McArthur W.P. (1978). Partial characterisation and the distribution of chicken mononuclear cells bearing the Fc receptor. J. Immunol. **120**: 1014-1020.

Gabe M. (1968). Techniques Histologiques (Paris: Masson et Cie).

Guillemot F.P., Oliver P.D., Peault B.M. and Le Douarin N. (1984). Cells expressing Ia antigens in the avian thymus. J. Exp. Med. **160**: 1803-1819.

Houldsworth J., Mathew S., Rao P.H., Dyomina K., Louie C.D., Parsa N., Offit K. and Chaganti R.S.K. (1996). *REL* proto-oncogene is frequently amplified in extranodal diffuse large cell lymphoma. Blood **87**: 25-29.

Hrdlickova R., Nehyba J. and Humphries E.H. (1994). v-*rel* induces expression of three avian immunoregulatory surface receptors more efficiently than c-*rel*. J. Virol. **68**: 308-319.

Hrdlickova R., Nehyba J., Roy A., Humphries E.H. and Bose H.R., Jr. (1995). The relocalization of v-*rel* from the nucleus to the cytoplasm coincides with induction of expression of Ikba and nfkbl and stabilization of I$\kappa$B-$\alpha$. J. Virol. **69**: 403-413.

Huguet C., Enrietto P., Vandenbunder B. and Abbadie C. (1994). C-Rel: A multifunctional transcription factor? Cell Death Diff. **1**: 71–76

Joos S., Otano-Joos M.I., Ziegler S., Brüderlein S., du Manoir S., Bentz M., Möller P. and Lichter P. (1996). Primary mediastinal (thymic) B-cell lymphoma is characterized by gains of chromosomal material including 9p and amplification of the *REL* gene. Blood **87**: 1571-1578.

Jotereau F.V. and Le Douarin N.M. (1982). Demonstration of a cyclic renewal of the lymphocyte precursor cells in the quail thymus during embryonic and perinatal life. J. Immunol. **129**: 1869-1877.

Kerr L.D., Inoue J., Davis N., Link E., Baeuerle P.A., Bose H.J. and Verma I.M. (1991). The rel-associated pp40 protein prevents DNA binding of Rel and NF-kappa B: Relationship with I kappa B beta and regulation by phosphorylation. Genes Dev. **5**: 1464-1476.

Kochel T., Mushinski J.F. and Rice N.R. (1991). The *v-Rel* and *c-Rel* proteins exist in high molecular weight complexes in avian and murine cells. Oncogene **6**: 615-626.

Köntgen F., Grumont R.J., Strasser A., Metcalf D., Li R., Tarlinton D. and Gerondakis S. (1995). Mice lacking the c-*rel* proto-oncogene exhibit defects in lymphocyte proliferation, humoral immunity, and interleukin-2 expression. Genes Dev. **9**: 1965-1977.

Kopp E.B. and Ghosh S. (1995). NF-$\kappa$B and Rel proteins in innate immunity. Adv. Immunol. **58**: 1-27.

Kralova J., Schatzle J.D., Bargmann W. and Bose H.R.J. (1994). Transformation of avian fibroblasts overexpressing the c-*rel* proto-oncogene and a variant of c-*rel* lacking 40 C-terminal amino acids. J. Virol. **68**: 2073-2083.

Le Douarin N.M. and Jotereau F.V. (1975). Tracing of cells of the avian thymus through embryonic life in interspecific chimeras. J. Exp. Med. **142**: 17-40.

Lewis R.B., McClure J., Rup B., Niesel D.W., Garry R.F., Hoelzer J.D., Nazerian K.A. and Bose H.R. (1981). Avian reticuloendotheliosis virus: Identification of the hematopoietic target cell for transformation. Cell **25**: 421-431.

Lu D., Thompson J.D., Gorski G.K., Rice N.R., Mayer M.G. and Yunis J.J. (1991). Alterations at the rel locus in human lymphoma. Oncogene **6**: 1235-1241.

Miyamoto S., Chiao P.J. and Verma I.V. (1994). Enhanced IkBa degradation is responsible for constitutive NF-kB activity in mature murine B-cell lines. Mol. Cell. Biol. **14**: 3276-3282.

Miyamoto S. and Verma I.M. (1995). Rel/NF-$\kappa$B/I$\kappa$B story. Adv. Cancer Res. **66**: 255-292.

Moore B.E. and Bose H.R. (1989). Expression of the *c-rel* and *c-myc* proto-oncogenes in avian tissues. Oncogene **4**: 845-852.

Morrison L.E., Boehmelt G., Beug H. and Enrietto P.J. (1991). Expression of *v-rel* in a replication competent virus: Transformation and biochemical characterization. Oncogene **6**: 1657-1666.

Morrison L.E., Kabrun N., Mudri S., Hayman M.J. and Enrietto P.J. (1989). Viral *rel* and cellular *rel* associate with cellular proteins in transformed and normal cells. Oncogene **4**: 677-683.

Nossal G.J.V. (1994). Negative selection of lymphocytes. Cell **76**: 229-239.

Oliver P. and Le Douarin N.M. (1984). Avian thymic accessory cells. J. Immunol. **132**: 1748-1755.

Olson L.D. (1967). Histopathologic and hematologic changes in moribund stages of chicks infected with T-virus. Am. J. Vet. Res. **28**: 1501-1507.

Peck R., Murthy K.K. and Vainio O. (1982). Expression of B-L (Ia-like) antigens on macrophages from chicken lymphoid organs. J. Immunol. **129**: 4-5.

Phi Van L. (1996). Transcriptional activation of the chicken lysozyme gene by NF-$\kappa$Bp65 (RelA) and c-Rel, but not by NF-$\kappa$Bp50. Biochem J. **313**: 39-44.

Quéva C., Ness S.A., Graf T., Vandenbunder B. and Stéhelin D. (1992). Expression patterns of *c-myb* and of *v-myb* induced myeloid-1 (*mim-1*) gene during the development of the chick embryo. Development **114**: 125-133.

Schatzle J.D., Kralova J. and Bose H.R. (1995). Avian I$\kappa$B$\alpha$ is transcriptionally induced by c-Rel and v-Rel with different kinetics. J. Virol. **69**: 5383-5390.

Sevoian M., Larose R.N. and Chamberlain D.M. (1964). Avian lymphomatosis. VI. A virus of unusual potency and pathogenicity. Avian Dis. **8**: 336-347.

Simek S. and Rice N.R. (1988). Detection and characterization of the protein encoded by the chicken *c-rel* protooncogene. Oncogene Res. **2**: 103-119.

Verma I.M., Stevenson J.K., Schwarz E.M., Van Antwerp D. and Miyamoto S. (1995). Rel/NF-$\kappa$B/I$\kappa$B family: Intimate tales of association and dissociation. Genes Dev. **9**: 2723-2735.

von Boehmer H. (1994). Positive selection of lymphocytes. Cell **76**: 219-228.

Weih F., Carrasco D. and Bravo R. (1994). Constitutive and inducible Rel/NF-$\kappa$B activities in mouse thymus and spleen. Oncogene **9**: 3289-3297.

Zhang J.Y., Olson W., Ewert D., Bargmann W. and Bose H.J. (1991). The v-rel oncogene of avian reticuloendotheliosis virus transforms immature and mature lymphoid cells of the B cell lineage in vitro. Virology **183**: 457-466.

